# Quality of free gingival graft content in youtube videos: Usability in patient information and student education

**DOI:** 10.4317/medoral.26011

**Published:** 2023-06-18

**Authors:** Selcen Ozcan Bulut, Nihan Ozel Ercel

**Affiliations:** 1 Nigde Omer Halisdemir University, Faculty of Dentistry, Periodontology Department, Turkey; 2 Mersin University, Department of Biostatistics and Medical Informatics, Turkey

## Abstract

**Background:**

The aim of our study is to evaluate the usability of the Free Gingival Graft (FGG) procedure, which is included in YouTube videos, in both patient information and student education.

**Material and Methods:**

A search was performed on YouTube on December 1, in 2022, using the search term ‘‘Free Gingival Graft’’. First 150 videos were pre-evaluated, and 67 videos were included in the study. The length of the videos, the number of views, the number of likes, the presence of animation and the number of months after uploading were evaluated. The quality of the videos was evaluated and analyzed with The Global Quality Score (GQS), Usefulness Score (US) and The Journal of American Medical Association (JAMA) scores.

**Results:**

A positive correlation was found between viewer interaction, video duration and quality scores. The median values of the quality scores were 2 for the GQS, 2 for the JAMA score and 1 for the Usefulness score. The level of quality scores was found to be insufficient (poor quality). There is a high level, positive and statistically significant correlation between the GQS and the Usefulness score (r=0.858 and *p*<0.001).

**Conclusions:**

YouTube videos containing the FGG procedure were found to be insufficient for both student education and patient information purposes.

** Key words:**Medical education, free gingival graft, patient information, social media, youtube.

## Introduction

Apical displacement of the gingival soft tissue margin from the enamel-cementum junction is defined as gingival recession and isa common clinical feature in the general population (1,2). Periodontal inflammation is involved in the etiology of gingival recession, but there are also predisposing factors that affect this inflammatory process (2-5). Periodontal inflammation may cause more significant bone loss and connective tissue destruction, especially in the presence of thinner buccal cortical bone (2). In the thin gingival biotype, there may be gingival recession due to plaque accumulation in areas where tooth cleaning is more difficult (2). In addition, the presence of thin and inadequate attached gingiva-keratinized gingiva, buccal displacement of the teeth, and trauma due to malocclusion are considered as predisposing factors (2,6). Incorrect toothbrushing can also cause gingival recession (7). Some studies argue that gingival health can be established with the presence of a minimum of 2 mm keratinized gingiva (6).

The main indications for root coverage procedures are aesthetic demands, treatment of tooth sensitivity, and enhancement of keratinized tissue to reduce the risk of defect progression (2,7). Gingival recession is divided into classes by Miller. This division determines the treatments to be applied according to these classes and also provides information about how much the root surface will be covered at the end of the treatment applied by the physician. Miller, based on his clinical experience, claimed that complete coverage of recession defects was feasible only for classes I and II, partial coverage was achievable for class III and no root coverage was possible for class IV (8). The treatment of gingival recession is possible with periodontal surgery, and it is very important whether the amount of attached gingiva is sufficient in terms of the prognosis of the treatment (8-10). While coronally advanced flap and/or connective tissue graft procedures, tunneling flap procedures are applied in the presence of adequate keratinized tissue for root coverage purposes, if this amount is insufficient, both root closure and an increase in keratinized gingiva can be aimed with the FGG procedure (2,11,12). In addition, other root coverage procedures can be applied after first applying FGG to the insufficient keratinized tissue and providing healing (9,10).

Although there are many soft tissue augmentation procedures to increase the width of keratinized tissue, FGG is the one of the most preferred procedures (13). Free gingival graft is a surgical procedure used especially for creating attached gingiva, as well as being used in root closure treatments (2). This procedure is basically based on the preparation of the recipient area, the removal of the graft from the donor area, and the placement of the graft on the recipient area, followed by feeding the graft from the recipient area and revascularizing it and integrating with the area (14,15). As with all surgical procedures, the FGG procedure should be able to know the indication, advantage, complication, prognosis of the procedures as well as the procedure to be applied by the physician.

YouTube is a very popular website that people easily access, thousands of new videos are uploaded every day and millions of videos are watched daily (16,17). Visual content can be scanned on almost any subject, including health-related topics, on YouTube (16-19).

In a study in the literature, 76.8% of individuals/patients declared that they use the internet for health purposes (20). Although patients use the Internet or YouTube to search for their medical condition, only 18% discuss this online search with their clinicians (21). However, YouTube is a site established for entertainment and social purposes, not for patient and student education, and there is no effective control mechanism for health education yet (19). In the literature, studies have started to evaluate the quality of some health applications of YouTube, but there is no study that evaluates the quality of videos related to FGG on YouTube in terms of education. In order to fill this gap, our study aimed to evaluate the usability of the FGG application in YouTube videos in both patient education and student education.

## Material and Methods

This study does not contain any human or animal resources, ethical approval was not needed for this study. Patient information was not used in the study. Therefore, the patient consent document was not obtained.

- YouTube Search and exclude criteria

**Figure 1:**
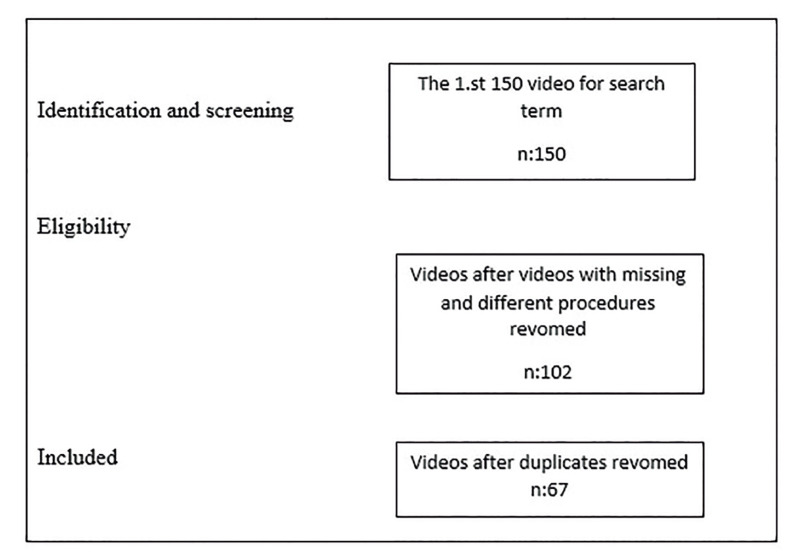
Search Strategy.

In this study, a search was performed on YouTube on December 1, 2022, using the search term ‘‘Free Gingival Graft’’. Based on the relevance to this keyword, the first 150 videos were recorded for assessment. Videos not related to the title, repetitive videos containing different procedures, and videos that do not contain all of the FGG procedures were not included in the study. The videos were watched by a single physician and their stated features were analyzed. Video length in seconds, view counts, number of likes, number of dislikes, video category (animation or not), video content, days since upload, and source of upload (uploader) were recorded. The usability of the videos in patient education was evaluated using the Global Quality Score (GQS) (22) criteria, and their usability in student education was evaluated using the Usefulness Score (US) (23). The quality of the videos was also evaluated with the Journal of American Medical Association (JAMA) scoring system (24,25). The 67 videos were analyzed according to the following criteria (Fig. 1).

- Quality Scores

JAMA scoring system (25): The JAMA scoring system is a non-specific and objective tool for online videos and resources. It consists of 4 individual criteria. Each criterion is scored 1 point and the total score ranges from 0 to 4 points. A score of 4 indicates high reliability and accuracy for the online source while a score of 0 indicates poor source reliability and accuracy.

Criteria Description:

Authorship; Author and contributor credentials and their affiliations should be provided.

Attribution; Clearly lists all copyright information and states references and sources for content.

Currency; Initial date of posted content and subsequent updates to content should be provided.

Disclosure; Conflicts of interest, funding, sponsorship, advertising, support, and video ownership should be fully disclosed.

Global quality score criteria (22): Non-specific educational content quality was assessed using the GQS (22). The GQS assesses the patient educational value of video content based on 5 criteria. The source is given 1 point for each of the present criteria. A score of 5 indicates the highest quality of education (22).

Score Description of quality:

Score 1. Poor quality; is unlikely of be to use for patient education.

Score 2. Poor quality; is of limited use to patients because only some information is present

Score 3. Suboptimal quality and flow; is somewhat useful to patients; important topics are missing, some information is present.

Score 4. Good quality and flow; useful to patients because most important topics are covered.

Sroce 5. Excellent quality and flow; is highly useful to patients.

The Usefullness Score for Students (23): Eight titles were evaluated, including Definition, indications, Contraindications, Advantages, Procedures involved, Complications, Postoperative, Prognosis and survival on YouTube videos and each content was scored as 1 point and the total score ranges from 0 to 8 points.

A score of 0 to 2 showed the content of poor [1] video that composed misleading information and was not all useful information about eight domains evaluated;

A score of 3 to 5 showed the content of moderate [2] video that gave a positive message related to FGG but poorly discussed some domains;

A score of 6 to 8 showed the content of excellent [3] video that gave detailed, valid and correct information for students recorded a usefulness score.

The usefulness score was determined based on quality and flow of the content;

poor [1]: poor quality, poor flow, missing and inadequate information.

moderate [2]: moderate quality, suboptimal flow, adequate information about content.

excellent [3]: excellent quality and flow, comprehensive and very useful information.

All data from 67 videos were statistically analyzed.

- Statistical analysis

Data analysis was performed with IBM SPSS Statistics Version 26 package program. The conformity of the data to the normal distribution was tested with the Kolmogorov Smirnov test of normality. Descriptive statistics of information about videos and scores are given as number (n), percentage (%), minimum value, maximum value and median (Q1-Q3). Spearman correlation test was used to determine the relationship between non-normally distributed video feature measurements and scores. The Kruskal Wallis test was used to compare the GQS and JAMA score medians according to the Usefullness classification, which has three categories. The Mann Whitneu U test was used to compare the medians of the score and video characteristics according to their genre, anime or not, doctor or not. All the results obtained were considered statistically significant at *p*<0.05.

## Results

67 videos were included in the study. 49 of these videos were uploaded by a doctor. While 9 of the videos are animated, 58 of them are not animated, and SDG surgery is shown on patients. Descriptive statistics of video-related features and quality scores are given in Table 1. The median values of the quality scores were 2 points for the GQS, 2 points for the JAMA and 1 point for the Usefulness (Table 1)

Correlations between video features and Quality scores are given in Table 2. There is a high, positive and statistically significant correlation between the number of video views and the number of likes (r=0.834 and *p*<0.001). Similarly, there is a high, positive and statistically significant correlation between the number of video views and the rate of viewing (r=0.879 and *p*<0.001). Considering the number of video views and the viewer interaction, there is a low, negative and statistically significant correlation (r=-0.376 and *p*=0.002). There is a high, positive and statistically significant correlation between the number of likes and the view rate (r=0.896 and *p*<0.001). There is a weak, positive and statistically significant correlation between the number of likes and the duration of the video (r=0.274 and *p*=0.005).

There is a weak, positive and statistically significant correlation between viewer interaction and video duration (r=0.457 and *p*<0.001). There is also a weak, positive and statistically significant correlation between viewer interaction and GQS (r=0.365 and *p*=0.002). In addition, there is a weak, positive and statistically significant correlation between viewer interaction and Usefulness score (r=0.301 and *p*=0.013).

There was a moderate, positive and statistically significant correlation between video duration and GQS (r=0.528 and *p*<0.001). There was a weak, positive and statistically significant correlation between video duration and JAMA score (r=0.431 and *p*<0.001). There is a moderate, positive and statistically significant correlation between video duration and usefulness score (r=0.509 and *p*<0.001).

Correlations between Quality Scores are given in Table 3. There is a weak, positive and statistically significant correlation between GQS and JAMA score (r=0.392 and *p*=0.001). There is a high level, positive and statistically significant correlation between the GQS and the usefulness score (r=0.858 and *p*<0.001). There is a weak, positive and statistically significant correlation between JAMA score and usefulness score (r=0.391 and *p*=0.001).

There is a statistically significant difference in terms of GQS medians and JAMA score medians between the poor, moderate, and excellent classes of Usefulness (*p*<0.001).

While there was a statistically significant difference between the animated and non-animated videos in terms of the number of views, medians of viewing rates and JAMA score medians, there was no statistical difference in other features and quality scores (Table 4).

While a statistically significant difference was found between the medians of the number of views and the medians of the JAMA score between the video source and non-doctor, there was no significant difference between the other features (Table 4).

**Table 1:**
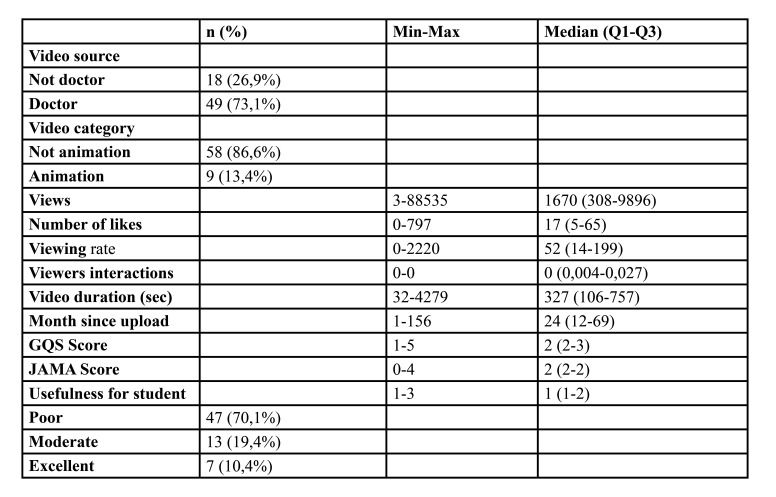
Descriptive statistics of features and quality scores for videos.

**Table 2:**
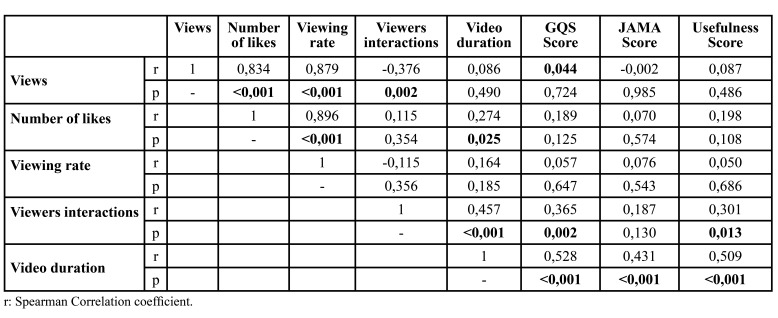
Correlations between video features and Quality scores.

**Table 3:**
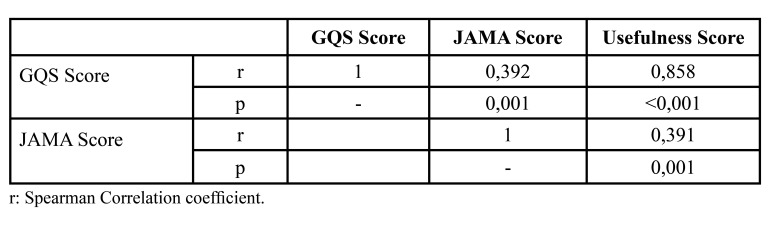
Correlations between Quality Scores.

**Table 4:**
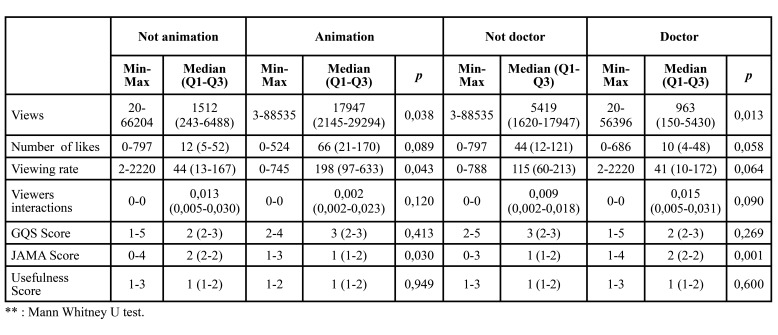
Comparison of scores according to whether the video is animated or not; and comparison of video-related features by source of video.

## Discussion

The internet is an easily accessible resource for health services and a comprehensive source of information (16,18). In this context, YouTube can also provide a lot of information for students and patients (17,19,26). In order to obtain information about the patients before the operation, dentistry faculty students also benefit from YouTube videos for visual purposes in education. Some researchers especially encourage medical students to use YouTube in this context (26). YouTube's various clinical approaches in learning anatomy, diagnosis and treatment of diseases, surgical methods, basic life saving methods, methods of protection from infectious diseases (17,19,26,27). Its usability in education in the field of education has been investigated (19,26). Patients can also frequently use the internet to learn about the procedures to be performed in gingival recession. Free gingival graft, which is one of the treatments used in gingival recession, is among the methods frequently preferred by physicians according to appropriate cases (2,10). Patients often want to investigate the treatment offered to them. YouTube, which has become widespread in health content in recent years, is a site that patients can access easily and has no cost to use (18,19). Patients and students frequently search from this site (26). In a study investigating the effects of YouTube videos on students' preferences and perception in the literature, concluded that the use of YouTube positively affects the education and training process (26). In yet another study, Azer *et al*. compared the information about cardiovascular mechanism in textbook and YouTube videos and showed that using YouTube can be ideal as a textbook (28). In our study, it was aimed to evaluate the usability of YouTube videos in Periodontology, and quality scores were used to measure the quality of the content related to the FGG operation, which is a mucogingival surgery, both in patient information and student education.

In the current study, in terms of content, 60% of YouTube videos were found insufficient for patient education, 70% for student education, and the quality of videos on YouTube was found to be insufficient for use for these purposes. When the literature is examined; Similar to our study, in an article investigating the quality of dental implants as content on YouTube, it was determined that YouTube has very limited quality (29). YouTube videos are related to the content searched and whether they offer quality and sufficient information varies (17,26,29). It should be checked whether YouTube videos provide sufficient and quality information for use in patient information or student education, especially in health-related content (19,23,30).

In our study, it was observed that the scores measuring the video quality increased significantly as the duration of the videos increased. In a previous study, it was seen that the video length was effective in the decision to watch or not watch the video (26). For this reason, it is very important for physicians/ medical educators to guide individuals/students (27,30).

In our study, it was observed that there was a positive and significant relationship between the video duration of the viewer interaction, GQS and US scores. In other words, the level of likes of the watched video was determined to be related to the quality of the video (25). The positive relationship between GQS and JAMA and US is an indication of the compatibility of the quality scales. As the usability level of the video in patient information increases, the usability level in student education also increases. However, there is also a statistical difference between the GQS and US scores, which supports the need to use videos that offer more comprehensive content in student education. Videos uploaded by a doctor often affects the JAMA score because the doctor in the video performs the operation; thus, it is seen that the number of views is high in the videos uploaded by a doctor and non-animated videos. In other words it is possible to say that source of the video, doctor or not, and whether it is animated or not are important factors for watching preferences of the viewers.

It is important to indicate for that there are some limitations in our study. As one of them, YouTube is a dynamic platform and the results have the potential to change depending on the upload of new videos in different time zones. Another limitation is that the videos that will be met by individuals when they search YouTube using other expressions other than the word "free gingival graft" may be different.

## Conclusions

Students and patients frequently use technological opportunities today, but technology offers many advantages when it is used correctly. Although the use of YouTube videos has advantages such as being cheap and easy, its use in student education and patient information is not sufficient for FGG, which is a periodontal surgery option.
